# Supplementary material for: Survived the Glaciations, Will They Survive the Fish? Allochthonous Ichthyofauna and Alpine Endemic Newts: A Road Map for a Conservation Strategy
Source: Animals (Basel). 2023 Feb 27;13(5):871. doi: 10.3390/ani13050871 (PMC10000147; doi:10.3390/ani13050871)
Supplement: Supplementary file 1 [file animals-13-00871-s001.zip › animals-2222516-supplementary.pdf]

Supplementary materials

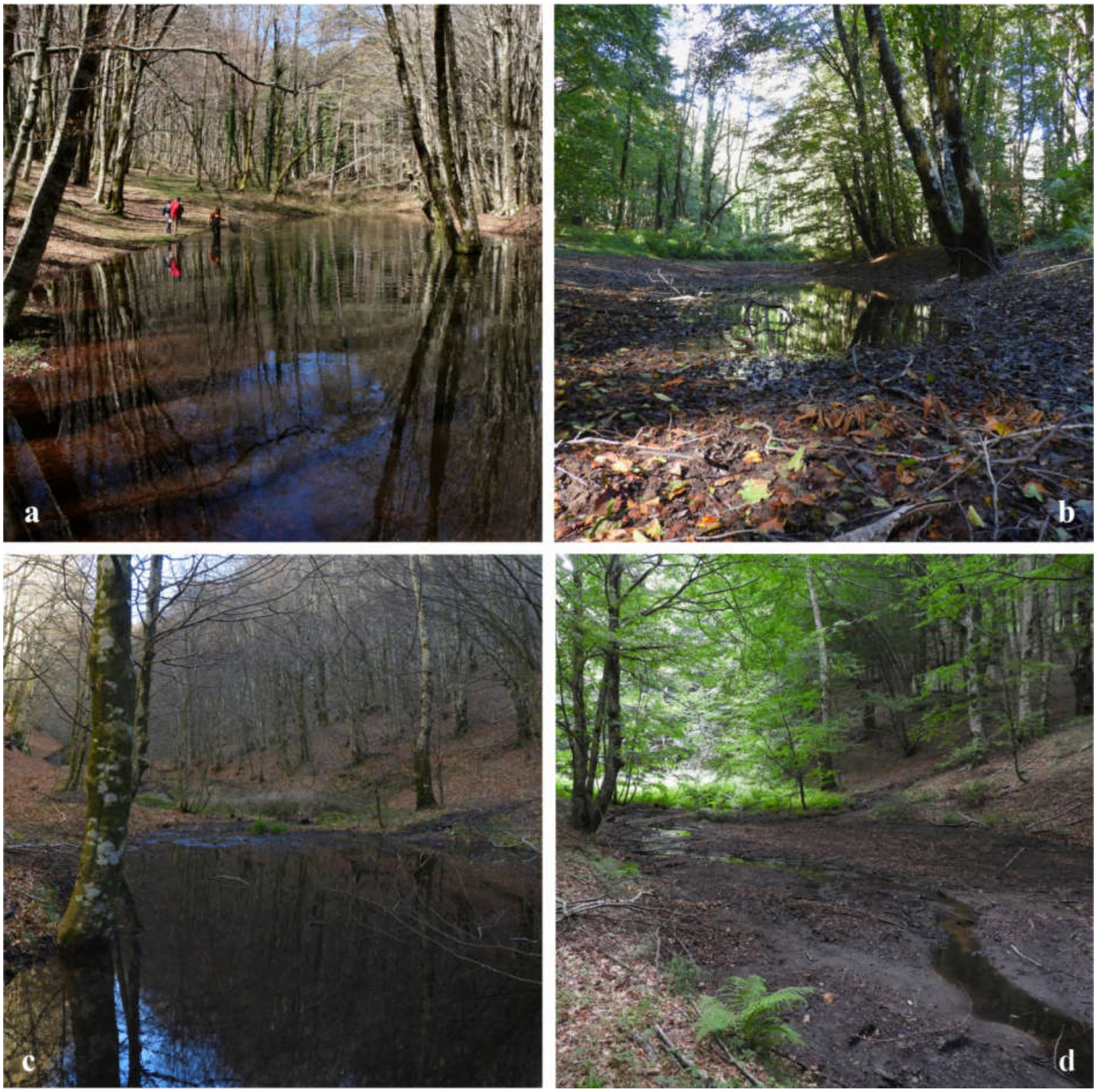

Figure S1. Representative images of the new breeding sites of *Ichthyosaura alpestris inexpectata*: Piano di Zanche in April (**a**) and September (**b**) 2022; Pantano Lungo in April (**c**) and July (**d**) 2022.

Table S1. Water physical-chemical parameters measured in the study ponds. Mean (±SD) of electric conductivity = EC, pH, total dissolved solids = TDS, oxygen percentage saturation = O2 %, dissolved oxygen = DO recorded in (s) summer and (a) autumn is shown.

	Site name													
	FO		DU		TR		TRIF		FA		PZ		PL	
**Parameter**	s	a	s	a	s	a	s	a	s	a	s	a	s	a
**T (C°)**	25.7 ± 0.6	13.7 ± 0.2	26.1 ± 0.2	18.8 ± 0.2	24.7 ± 0.6	12.8 ± 0.2	23.5 ± 0.5	12.6 ± 0.2	26.1 ± 0.3	18.8 ± 0.3	19.2 ± 0.4	13.2 ± 0.4	15.9 ± 0.4	12.6 ± 0.4
**EC (µS/cm)**	84.3 ± 0.6	78.3 ± 1.5	58 ± 1	40.3 ± 0.6	78.3 ± 0.6	72.7 ± 0.6	78 ± 1	80.7 ± 0.6	81 ± 2	82.5 ± 0.6	88.8 ± 0.6	85.2 ± 0.3	81.7 ± 1.1	98.3 ± 0.6
**pH**	6.3 ± 0.06.	6.6 ± 0.05	7.2 ± 0.1	6 ± 0.05	6.7 ± 0.1	5.9 ± 0.02	6.8 ± 0.05	6 ± 0.05	6.5 ± 0.06	6.2 ± 0.1	6.1 ± 0.05	6.5 ± 0.1	6.9 ± 0.05	6.6 ± 0.09
**TDS (ppm)**	43.1 ± 0.5	38.8 ± 0.6	31.2 ± 0.5	25.4 ± 0.4	39 ± 0.5	36.3 ± 0.6	39.2 ± 1	46.3 ± 0.5	40 ± 0.5	42 ± 1	45.4 ± 0.4	48 ± 0.6	40.2 ± 0.2	50.4 ± 0.2
**O_2 %**	65 ± 1	72 ± 1	70.1 ± 0.5	75 ± 0.6	68 ± 1	78 ± 0.5	65.2 ± 0.2	70 ± 0.7	61.2 ± 0.2	68 ± 0.5	46 ± 0.3	46 ± 0.2	58.1 ± 1	65 ± 0.5
**DO (mg/L)**	8.8 ± 0.06	9.8 ± 0.02	8.8 ± 0.01	8.7 ± 0.3	7.8 ± 0.6	11.3 ± 0.03	5 ± 0.02	8.9 ± 0.01	8.5 ± 0.05	8.9 ± 0.05	5.2 ± 0.02	5.4 ± 0.01	6.8 ± 0.03	6.9 ± 0.06

Table S2. Taxonomic list and specimens abundance (number of individuals m^{-2}) relative to the total of 43 Families, 15 Orders, and 7 Classes of macroinvertebrates collected in the two sampling campaigns involving the four selected monitoring sites. June = J, October = O, TRIF = Trifoglietti inferiore, PL = Pantano lungo, PZ = Piano di Zanche, FA = Fossa Armando.

Taxa			TRIF		PL		PZ		FA	
**Class**	**Order**	**Family**	**J**	**O**	**J**	**O**	**J**	**O**	**J**	**O**
Arachnida	Hydrachnida	Hydrachnidae	11	-	-	-	5	5	16	6
Bivalvia	Eulamellibranchi	Sphaeriidae	219	475	149	421	-	133	80	-
Crustacea	Isopoda	Asellidae	144	112	-	80	-	-	144	16
Gordioida	Gordioidea	Gordiidae	-	-	5	-	-	11	-	-
Hirudinea	Hirudinida	Haemopidae	27	11	-	-	-	-	48	-
Insecta	Plecoptera	Capniidae	-	-	85	16	-	-	-	-
		Leuctridae	-	-	112	16	-	-	-	-
		Nemouridae	-	-	21	21	-	-	-	-
	Ephemeroptera	Baetidae	90	165	37	-	-	-	160	-
		Heptageniidae	-	-	-	11	-	-	-	-
	Trichoptera	Helicopsychidae	-	-	5	-	-	-	-	-
		Hydropsychidae	-	-	27	-	-	-	-	-
		Lepidostomatidae	-	-	-	-	37	32	-	-
		Limnephilidae	27	272	240	133	27	314	-	139
		Odontoceridae	-	-	112	75	32	112	-	85
		Phryganeidae	-	-	-	-	11	11	27	27
		Sericostomatidae	283	192	91	43	373	608	442	-
	Diptera	Ceratopogonidae	37	-	37	-	-	-	21	16
		Chironomidae	619	405	715	459	229	53	885	-
		Culicidae	-	-	-	-	155	133	-	-
		Dixidae	-	-	32	5	-	-	-	-
		Limoniidae	-	-	16	21	-	-	5	-
		Simuliidae	-	-	96	-	-	-	-	-
		Tabanidae	-	-	5	-	-	-	5	59
	Odonata	Aeschnidae	43	32	5	27	-	-	-	-
		Calopterygidae	-	5	-	5	11	-	-	11
		Coenagrionidae	-	37	-	-	-	-	-	-
		Cordulegasteridae	-	-	3	21	-	-	-	-
		Libellulidae	5	11	-	5	-	-	-	5
	Coleoptera	Dryopidae	5	-	-	-	-	-	21	-
		Dytiscidae	-	21	-	53	-	-	48	48
		Elminthidae	-	-	-	-	-	-	-	11
		Gyrinidae	5	-	-	-	-	-	5	-
		Haliplidae	-	11	5	48	-	-	-	11
		Scirtidae	-	-	37	59	283	251	-	-
		Hydraenidae	-	11	-	-	11	16	-	11
		Hydrophilidae	-	-	-	-	-	-	5	-
	Heteroptera	Nepidae	-	-	-	5	-	-	-	-
		Notonectidae	5	-	-	5	-	-	43	-
		Veliidae	-	-	5	5	-	-	-	-
Oligochaeta	Opisthopora	Lumbricidae	-	5	21	21	-	-	5	5
	Lumbriculida	Lumbriculidae	11	11	-	37	-	-	-	229
	Tubificida	Naididae	27	16	5	16	-	-	11	-
**Total**			1558	1792	1866	1597	1174	1679	1971	743

Table S3. Morphometric parameters (SVL: Snout vent length; SMI: Scaled mass index) of *Ichthyosaura alpestris inexpectata* from the study sites (TRIF: Trifoglieti inferiore; FA: Fosso Armando; PZ: Piano di Zanche; PL: Pantano Lungo).

Site	Sex	N	Body mass (g)		SVL (mm)		Total length (mm)		SMI	
			mean ± SD	range	mean ± SD	Range	mean ± SD	range	mean ± SD	range
TRIF	M	24	2.4 ± 0.49	1.6 - 3.3	47 ± 4.13	40 – 57	81.29 ± 5.4	71 - 90	2.41 ± 0.44	1.46 - 3
	F	18	4.14 ± 0.94	2.5 - 5.7	53.5 ± 3.20	48 – 60	93.5 ± 5.43	85 - 103	4.14 ± 0.76	2.41 – 5.45
FA	M	11	2.66 ± 0.32	2.1 - 3.1	45.94 ± 2.93	43 – 51	77.91 ± 3.96	73 - 86	2.72 ± 0.35	2.07 – 3.35
	F	4	4.57 ± 0.74	3.9 - 5.4	54.5 ± 3.32	50 – 57	93 ± 5.48	85 - 97	4.6 ± 0.77	3.47 – 5.12
PZ	M	5	3.54 ± 0.24	3.2 - 3.8	48.8 ± 1.09	48 – 50	83.8 ± 2.17	80 - 85	3.54 ± 0.16	3.37 – 3.79
	F	4	4.87 ± 1.22	3.8 - 6.5	53.25 ± 2.75	50 – 56	94.25 ± 6.75	87 - 100	4.85 ± 0.67	4.04 – 5.59
PL	M	16	2.71 ± 0.41	2.2 - 3.6	47.19 ± 3.54	40 – 54	81.06 ± 6.27	70 - 93	2.71 ± 0.34	2.3 – 3.35
	F	42	4.42 ± 1.24	2.2 - 7	52.74 ± 4.82	40 – 60	95.55 ± 5.68	85 - 105	4.35 ± 0.73	3.17 – 6.19
